# Adapting an Evidence-Based Intervention Targeting HIV-Infected Prisoners in Malaysia

**DOI:** 10.1155/2011/131045

**Published:** 2011-08-15

**Authors:** Michael M. Copenhaver, Noor Tunku, Ifeoma Ezeabogu, Jessica Potrepka, Muhammad Muhsin A. Zahari, Adeeba Kamarulzaman, Frederick L. Altice

**Affiliations:** ^1^Department of Allied Health Sciences, University of Connecticut, 358 Mansfield Road, Box U-2101, Storrs, CT 06269-2101, USA; ^2^CERiA, University of Malaya, 50603 Kuala Lumpur, Malaysia; ^3^Department of Psychological Medicine, Faculty of Medicine, University of Malaya, 50603 Kuala Lumpur, Malaysia; ^4^Section of Infectious Diseases, Department of Medicine, Yale School of Medicine, Yale University, New Haven, CT 06510-2283, USA

## Abstract

HIV-infected prisoners in Malaysia represent a critical target population for secondary HIV risk reduction interventions and care. We report on the process and outcome of our formative research aimed at systematically selecting and adapting an EBI designed to reduce secondary HIV risk and improve adherence to antiretroviral therapy among soon-to-be-released HIV-infected prisoners. Our formative work involved a critical examination of established EBIs and associated published reports complemented by data elicited through structured interviews and focus groups with key stakeholders, members of the target population, and their family members. Based on all information, we adapted the Holistic Health Recovery Program targeting people living with HIV (HHRP+), an EBI, to consist of eight 2-hour sessions that cover a range of specified topics so that participants may individually apply intervention content as needed to accommodate their particular substance abuse, HIV risk, and antiretroviral adherence issues. This study provides a complete example of the process of selecting and adapting an EBI—taking into account both empirical evidence and input from target organization stakeholders and target population members and their families—for use in real world prison settings where high-risk populations are concentrated.

## 1. Introduction

HIV-infected drug users who cycle into and out of correctional systems continue to fuel the HIV pandemic and, as such, they represent a significant vector for HIV transmission worldwide. Certain parts of the world, such as Malaysia, are disproportionately impacted by this problem [1]. A rapidly expanding body of research literature, including that of our own international team of investigators, indicates that criminal justice facilities can be ideal settings for identifying previously undiagnosed cases of HIV [2–6] as well as initiating treatment for HIV [2, 7–9]. Providing secondary HIV risk reduction interventions and engaging inmates in subsequent care, however, is often challenging [10–12] and insufficient [13–15].

Because reentering the community upon release is often characterized by a significant decline in all forms of healthcare for HIV-infected prisoners, this transition can represent a grave threat to individual and public health. A key issue facing the correctional and community healthcare systems today is therefore how to best assist inmates to maintain the low HIV transmission risk status they have been able to achieve as they transition from the highly structured prison setting [16]. 

Evidence-based interventions (EBIs) have been developed for HIV-infected drug users in prisons [3] as well as drug users enrolled in community-based drug treatment settings [17, 18], though none to date have been successfully tailored to prepare inmates for the extremely high risk period immediately following release during which newly released HIV-infected prisoners are becoming reintegrated into the community. Our research team has addressed this unmet need by conducting formative research designed to inform the selection, adaptation, and placement of an evidence-based secondary HIV risk reduction and antiretroviral adherence intervention specifically for implementation during the transition period when HIV-infected prisoners are preparing to return to the community. The complete process and our findings, including the adapted intervention, are outlined below.

## 2. Methods

### 2.1. Formative Research

We prepared for the implementation of an HIV risk reduction intervention among drug-involved soon-to-be-released HIV-infected prisoners by conducting formative research that first involved reviewing the available evidence-based interventions (EBI; see http://www.effectiveinterventions.org/), and then selecting and adapting an EBI, as needed, for implementation among the target participants in Malaysia. Thus, we sought to identify an EBI designed for drug-involved HIV-infected persons that could be readily adapted for use with soon-to-be-released HIV-infected prisoners in Malaysia.

#### 2.1.1. Selecting an EBI

As in prior studies [19, 20] our first step was to review all of the EBIs (see http://www.effectiveinterventions.org/) that were applicable to the target population. An EBI was considered applicable to our target population if was designed for persons characterized as (1) HIV-infected, (2) having a preincarceration history of opioid dependence, and (3) seeking drug treatment upon release from incarceration. Second, we rank-ordered the selected EBIs based on the extent to which the intervention (1) included content designed to address sex-risk, drug-risk, and antiretroviral adherence behavior, (2) was theory-driven, (3) had been implemented with a range of relevant ethnicities/populations, and (4) had demonstrated feasibility to be adapted for implementation in relevant clinical contexts. Using this two-tiered evaluation approach, we concluded that the Holistic Health Recovery Program for HIV-infected persons (HHRP+ [21]) would serve as the optimal foundation for our intervention approach targeting drug-involved HIV-infected prisoners in Malaysia.

### 2.2. Adapting an EBI

Because our ultimate objective was to implement our intervention in a novel cultural context, it was critical to take into account a host of factors relating to language, customs, and social norms in tailoring our intervention approach to accommodate the target population. Thus, we incorporated the framework below for this purpose.

#### 2.2.1. Global Framework: Accommodation, Incorporation, and Adaptation

First, in order to develop a culturally responsive intervention targeting HIV-infected prisoners in Malaysia, our overarching approach involved drawing upon Wiley's framework—which includes accommodation, incorporation, and adaptation—as the three initial courses of action for working with the target population [22]. *Accommodation* required a keen understanding of the communication styles and literacy practices among the target participants and to taking such factors into account in developing the intervention content and delivery style. Because our research team included several native Malaysians with significant experience conducting community-based intervention projects with the target population in Malaysia, we were readily able to engage in this course of action throughout the intervention development process. *Incorporation *required our team to become acquainted with community practices customs and to incorporate these directly into the intervention approach. Promoters of this perspective emphasize that community constituents (such as drug treatment programs, community-based organizations, correctional settings, and families) must fully understand target participants and adjust intervention content and delivery to address their needs. For the purposes of our study, *Adaptation *involved approaching the intervention development process with the philosophy that, in addition to conveying specific information and skills to reduce health risks, intervention content should also promote target participants' adjustment to prosocial norms of the communities to which they are preparing to return [22].

#### 2.2.2. Elicitation Research

Next, based on the Assessment-Decision-Administration-Production-Topical experts-Integration-Training-Testing (ADAPT-ITT) model of intervention adaptation [23], we sought to collect information from members of the target population, their family members, and treatment providers and other stakeholders in the target organizations (i.e., correctional and community treatment settings) where the intervention was expected to be implemented (Tables 1 and 2). The objective of conducting interviews with members of the target population was to assess their baseline knowledge and attitudes about HIV transmission and risk reduction strategies in order to properly adapt our intervention. We also sought to understand their attitudes about methadone maintenance therapy as part of a risk reduction strategy and to gain their input about what type of risk reduction intervention approach they felt would be most helpful. Because we were aware of the relative importance placed upon family relationships in Malaysian culture, as well as the high degree of involvement that family members were expected to play in terms of supporting inmates upon release, we conducted separate interviews with any family members who were willing to participate. 

The objective of the interviews with the treatment providers was to assess their current practices involving the target population in terms of HIV risk reduction and to understand the challenges they currently face in that regard. We also wanted their input in designing and implementing an intervention that would have a strong likelihood of acceptance and success within their prison settings. The prison counselors were also interviewed to assess their perception of their patients' knowledge of HIV as well as their attitude towards sex- and drug-related risk reduction. Since the counselors were viewed as the most likely be directly involved in the delivery of a risk reduction program, we also questioned them about their ongoing risk-reduction-related efforts and challenges as well as querying them about what they thought would be needed to make an intervention work best. Our objective in interviewing correctional officers was to assess their perception of the methadone program as well as getting their input about the design of a risk reduction intervention. The correctional officer participants were also able to provide detailed information about what materials could be brought into the prisons and other logistics that should be considered when planning to conduct such an intervention.

**(1) Participants**

We sought to interview recently released prisoners (and families) who shared key characteristics with our target population (e.g., HIV-infection, history of injection drug use, history of drug-related incarceration). We conducted semistructured interviews with recently released drug-involved HIV-infected prisoners in Malaysia (*n* = 8) as well as their family members (*n* = 3). All the recently released prisoners were males and frequency of incarceration ranged form 2 to 10 times and length of incarceration ranged from 2 months to 1 year. Only one of the recently released prisoners mentioned having a wife. During the same time frame, we conducted focus groups with prison physicians (*n* = 5), prison counselors (*n* = 8), and prison correctional officers (*n* = 4) from several prison facilities throughout Malaysia (Table 3). Our objective was to interview treatment providers in correctional facilities involved in assisting HIV-infected inmates with their HIV-focused healthcare while incarcerated.

**(2) Procedure**

As in prior work [19], we used structured but open-ended instruments to obtain interview data (Tables 1 and 2). All participants were informed that the objective was to elicit a range of information that could collectively guide the development of an HIV-focused risk reduction program that could be implemented during the transition period in which inmates were being released to the community. Thus, some items focused on the relevant characteristics of the target population while other items focused on ways to optimize intervention content, delivery, and placement within a facility.

All interviews and focus group sessions were audiotape recorded and transcribed in Bhasa and translated to English. The English translation was backtranslated to Bhasa to ensure appropriate translation. Trained Bachelor's and Master's level researchers conducted the interviews and focus group sessions as well as the content analysis of the data [24] under the supervision of a licensed clinical psychologist. For the content analysis, responses to each item were grouped and summarized by two master's level graduate assistants and results were compared for any inconsistencies. The primary themes identified formed the basis of the selection of an appropriate intervention and the subsequent adaptation of the intervention to meet the specific needs of the target population. Based on content analyses performed, the primary themes were identified and are summarized in the section below. The study protocol was approved by Yale University's Human Investigation Committee and the University of Malaya's Ethics Committee.

## 3. Results

### 3.1. Interviews with Target Population and Family Members

**Primary Issues Faced When Released**

When asked to describe the key concerns they had while preparing for release, typical responses from the prisoners included

*“My plan when I got out… I didn't have any plans. Because that time when I was relapsed, then I was confirmed that I got TB. When I got cured from TB, I didn't plan for anything. I just planned to quit this drug taking, didn't want to use any drugs, didn't want to become a burden to my parents. My concern? Relapsing, smoking heroin”* and *“one of the reason is that la… using the drug back, Afraid that you will be like before? Everything will be haywire. My life before… in simple terms… I… when I was taking drugs… and when I was taking tablets… one day I can… spend up to hundreds of ringgit… until I am unconscious. Sometimes for days. I take tablets… I injected.” *

When asked how soon they resumed drug use after their release, responses included

*“… the same day”* and “*Not long… not even up to a week.” *

Half of the participants reported they had injected heroin upon release and that it was typical to do so within their first week of release.

Family members all reported that they were aware of their relative's HIV-positive status and had heard it directly from the relative. Primary concerns among family members when their relative was released are exemplified by these responses:

“*… I think it was more of a hope that he can [still] have a very long life, but I know that is difficult because of his condition”, *and* “… you know, with society, when you have something like [HIV], people won't… don't want you to get married.” *

When asked specifically about their willingness to provide long-term support to their relative and if they had placed any conditions (e.g., sobriety) on their continuing to live at home following release from prison, some of the responses were

*“She [the mother]… is never really like… set in stone conditions. But she does advise him… like you know, you got to change and all of that… she always takes him in… she's never… never said no”* and *“No, no, no… no restrictions, nothing….” *

**Knowledge about HIV Transmission**

With regard to gains in HIV-related knowledge while in prison, one participant reported

*“All we know already… in prison all they… they give ah… what… like brochures… All sorts…. There is [peer] counseling… for the new inmates….”*

and all who responded agreed that they were provided with helpful information. When asked about the key modes of HIV transmission, typical responses included

*“Injecting needles… sharing…, Having sex with a woman” *and* “There are three groups right? One who are drug users, one who is like… men (and) women… and the other is from a mother to her child*.*” *

Several participants indicated that they believed HIV could be transmitted via saliva, shared cooking and eating utensils, and by being too close to children. Further, several participants were unaware that HIV transmission can occur through sharing drug cookers although they knew that it could be transmitted by sharing syringes.

All family members indicated that they knew that HIV could be transmitted via unprotected sex and sharing needles. When asked if they feared for the health of other family members, one response was

*“Oh yes. In fact… first when we… we… ah… we found that he got HIV, we are scared to share…. In fact, we asked him to, if he is taking… taking his lunch or dinner, we ask him to… to use separate… ask him to eat first. (laughs). We eat… we eat later Ah, let's hope for the best. I leave it to God. Because… because at first, I am a little bit scared….”*

Two of the three reported that they were scared for the health of other family members after learning of their relative's HIV-positive status.

**Common HIV Risk Behaviors**

In the discussion about common types of HIV risk behaviors among prisoners, regarding condom use, one participant reported

*“… We don't have the allowance for (condoms) in prison… Can't do anything about it.”*

None of the participants had heard of using bleach to sterilize needles but one participant reported

*“No, because when we are around… we are cooking our drug… the pot like that… we put our syringe like that… and then we cook in there… the water's boiled. We take out, put our drugs and we cook it back.”*

All participants responded they knew it was important to not share needles, though only two participants reported never sharing needles. One participant reported

*“Yes, I have shared but with other HIV people also… with infected persons.”*

With regard to sexual behavior, most participants reported abstaining from sex since their release. Responses included

*“No. I only concentrate on my drugs only…”* and *“Because when I get that thing [heroin], I don't have the urge at all….” *

Of the participants who reported continuing to have sex and using condoms, one responded

*“I've used one, but not sure I'm using it right”*

while another indicated that he did not use condoms regularly. One of these participants added that, although condoms were good to use for safety, they greatly diminished sexual pleasure. The other sexually active participant stated [through the translator]

*“Because they are Muslims, they can't use condoms” *and* “For the singles, they aren't supposed to have sex… so they may [just] use drugs and not be too interested in sex.” *

**Methadone Maintenance as a Risk Reduction Strategy**

Seven of the eight participants were currently enrolled in methadone maintenance therapy and one of the eight was receiving suboxone (another opioid agonist therapy for treating opioid dependence). When asked about their experience with methadone, some of the responses were

*“Well, my physical [health] is normal…. I can do anything I want. I don't have to worry about money… or finding drugs…. I feel like I have become a normal person”, “Ah… before, it was really worse…. My… my drug addiction… [much] worse… but I got this methadone, now is very slow already. And I think I can ah… makework, I can slowly…. I can… like normal people… Can… can… can work… can work… that's why I think this methadone also is very… very good for me. I think I can go on”* and *“Ahh… not very effective. the suboxzone is better.” *

When asked how they felt about being on methadone for the long term, some responses were

*“[Methadone] will interrupt our life because we will need to show up at the clinic every day and that will make it difficult to stay in treatment”* and *“When we start working, it may be an obstacle to go to the clinic before work.” *

However, all participants reported that they had experienced and accepted the side effects of methadone—primarily sleepiness, constipation, itching, skin infection, vomiting, and diarrhea—as well as accepting the reality that they would likely need to be on methadone long-term as indicated by

*“Have to accept that reality*—*that we need to be on this for a long time to come.” *

Regarding their impression of the role of methadone, some responses of family members were

*“Now he seldom… goes out to meet his… friends and all. He stays at home… [laughs] … and is very much better. You can see some improvement in him” *and *“He said in his opinion, the methadone is really great, good, really helps [mentions son's name] … you know ahh… helps him a lot. He's improving a lot. But sometimes he said he notices that he ah… can't sleep very well.” *

Family members all reported being somewhat familiar with drug treatment therapies including methadone and had a generally positive impression of the potential role they can play in recovery. In particular, family members believed that this type of treatment helped with urges to return to prior drug-using behaviors. Two family members expressed interest in learning more about it.

**Intervention Content and Delivery**

When asked for ideas to help design an appropriate HIV risk reduction program, participants expressed that the target population would likely want to participate in a risk reduction type of intervention as exemplified by

*“Before… before… 1 month or 2 months… maybe good for… maybe effective… one month or 2 months before release….”*

When queried specifically about the mode of intervention delivery, participants tended to favor a group as opposed to a one-on-one session format.

With regard to learning about condom use, some responses were

*“Yes, it would be useful for those who are married and have a wife”* and *“Yes, it would be useful as well for [an unmarried prisoner].” *

It was also suggested that the families of prisoners need to know more about HIV and how it is and is not transmitted. When asked about the extent to which it would be helpful to have their family actually involved in the intervention, participants were divided as exemplified by the following responses

*“Most people who enter prisons, they are like me—hardcore. Their families wouldn't want to come… They are fed up with seeing us in the prisons. Anyway, it would be hard. They'd have to spend [a lot of money]” *and *“I think it would be better….” *

Participants also expressed that the usefulness of family involvement in the intervention content would greatly depend on the topic. For example when asked specifically if sexual behavior could be discussed with family around, one response was

*“Maybe for the married ones, with their wives, but not those who are still single, it wouldn't be appropriate.”*

Participants felt that it would be helpful to include family members in content related to methadone therapy and HIV medication management. All participants reported that their families were aware of their HIV status and all stated that they had returned, or were going to return to living with their family immediately upon release.

When family members were asked to describe the best way for someone to completely stop using drugs, participants responses included

*“…the most important thing I think is encouragement from the family. Don't just leave,… leave him… alone or on his own.”*

Family members all reported trying to help their relative by giving advice and seeking professional help as indicated by

*“… if he ask questions, if he asks… advice, we just give advice—say this drug is not… not good for you. We start asking questions—what is the best, ah… best method to… I mean to ask for help. And then we… the first thing, we saw the Professor….”*

and they reported feeling that these types of efforts had been fruitful. When they were asked if they would find HIV and methadone maintenance education helpful, their responses were positive:

*“Yes. To be frank,… I don't know anything…”* and *“I think yes. I want to…. I want to know more about…. I don't know… I don't exactly know what is really the methadone… ah… what is it called. Is it a type of medicine? Is it a drug or not? I'm not very sure….” *

However, when queried about whether they would be willing to go to the prison or treatment center to get some education, one participant responded

*“Ah… she said that ah it's [a good idea] … but you need to ensure that the family wants to do this since it might be difficult” *and another, *“I don't think so….” *

### 3.2. Interviews with Treatment Providers

#### 3.2.1. Interviews with Prison Physicians (*n* = 5)

**Patients' Knowledge about HIV and Risk Reduction**

When asked about their patients' level of knowledge about HIV transmission, one of the prison physician participants reported

*“Mostly, they know because HIV positive people already have sufficient knowledge about that. For myself, I used to do every three months from… and I present it. I choose one day for… that day no other prisoner can come into my place… and I talk to them…. I go through with them with their family not with the help of the Prison Department… because of people around, they very scared because they don't know that HIV never goes through from the saliva,… they all wear the mask and they all wear the gloves….”*

They all tended to agree that there was an adequate level of knowledge about HIV. Only one physician reported feeling that it was necessary to counsel his patients about the various types of HIV risk. Some reasons for choosing to not counsel patients in this regard were captured in this comment:

*“The problem… is the counselor. We have no communication with the counselors who are uniformed staff; we are non-uniformed. So that is a big big barrier. [When] we approach them, they think “who are you to tell met….” that sort of attitude. So there is a big communication gap with the counselors [and physicians] … forget the prisoners. They come for treatment—yes, I do see them—but if you think, I do talk to them counseling wise… Socially, I do not have the time, I do not have the manpower on my, in my clinic. I don't have that.”*

Common barriers were not having enough time, concern about confidentiality issues (i.e., they have no right to speak with prisoners in the absence of prison management), and an undesirable working relationship with the prison counselors who are primarily responsible for speaking with patients.

When asked why they believed their patients continue to practice risky behaviors such as drug use and sharing needles, example of the physicians' response is

*“Some of our patients, most of our clients are not staying with their family members, they move to stay with their friends. They… stay… around the area for the drugs”* and *“I talk to them and they give me the points. They smoke.” *

**Methadone Maintenance as a Risk Reduction Strategy**

When probed for their views about the use of methadone as part of a risk reduction program, an example of their response is

*“[When released from prison], they will definitely go back to whatever it is [their drug of choice], but if you can put them [on methadone], the relapse rate after release will probably be reduced by 50%.”*

Physicians also reported that their patients were quite enthusiastic about enrolling in methadone maintenance as exemplified by

*“When we started talking to them about methadone therapy and now I find every one of them comes to me and… give me a note. So I give a lot of notes to them saying that that if you are interested in the methadone therapy, go to the clinic, pharmacy clinic to get the treatment. So I think a lot.”*

Some potential challenges were noted, however, including the need for good communication across prison staff exemplified by this comment:

*“No In my opinion giving an administrational methadone is not a problem, on our part. The problem I feel the prison, is concerning the pre-methadone counseling where we are not involved at all. We don't know anything.”*

Finally, physicians also expressed concerns that there were too few methadone programs outside the prisons and that some patients would be faced with the decision to either travel long distances for treatment or to discontinue treatment.

**Antiretroviral Therapy following Release**

When asked about prisoners' ability to continue proper adherence to antiretroviral medications following release, some of their responses were

*“[If] you start them on methadone, it is not as much of a problem because they will definitely go look for a methadone program where it can be continued, but if he is on HAART and doesn't find family support when released, he is not going to be able to continue with the HAART. That's our worry”* and *“We… officer, prison doctor… as well as Health Department. Health Department is pushing this very much… for HAART treatment… because… HAART specialist… very very difficult to get an appointment with the HAART…. I can get the appointment… HAART treatment and the follow up, CD4 count and… so to get that… that is very hard.” *

The general opinion was that HIV management was very difficult inside and outside the prisons.

**Family Involvement**

When asked whether family involvement should be encouraged in a risk reduction program, some of their responses were

*“The only people who will accept them in Malaysia, I tell you, and in my opinion are the family. They are not welcomed by any other group” *and* “… but there are prisoners who tell you that they have nobody to go back to, nobody wants me and you see, I am going to be released… within the next few weeks, I will be back here. And the cycle goes on. They are released, and within a few weeks they are back.” *

Concerns about having families visit the prisons were raised as exemplified by

*“This is a very difficult situation. We can do that but some of them… [it is] very difficult to meet their family member”* and *“Because of their busy… security system… we cannot… sometimes it is very difficult to [do that].” *

One suggestion for supporting the prisoners after release was

*“Basically, we find prisoners… most of our HIV+ prisoners are drug addicts. So we have drug users, they either don't have family or nobody wants them, so they have nowhere to go. Even if you try to contact their family, the family doesn't want to take… them home, carry on. so what I am trying to do now is that, we feel that… HIV and HAART treatment itself like some of our patients have… so we have to find a half way house where they can go to some place where they will take care of them and continue with whatever treatment that we carry on and later if he is able to support himself then we carry on for… years.”*

**Intervention Content and Delivery**

The physician participants all agreed that conducting group sessions would be the most feasible option in terms of time constraints. When further discussing the details of the intervention content, one physician encouraged the use of videos clips and pictures, as exemplified by

*“Pictures, video, speaking in demonstrations… that's something that can be very helpful.”*

They stressed the need for a multidisciplinary strategy with everyone making a contribution, as captured by

*“I think from the start that everybody should be told that this is a multidisciplinary approach.”*

Physician participants also strongly agreed on several issues specific to patient care including the need to incorporate the concurrent use of methadone and antiretroviral medication for patients as exemplified by

*“… they have to go somewhere—otherwise it is very difficult to start them on the treatment. Otherwise… if you start them on methadone, is not much of a problem but they will definitely go look for methadone if you really find a place for them to go and get it. But if they go to Kajang and get HAART and then doesn't find family [after release], he is not going to go and continue with the HAART. That's our worry.”*

They emphasized the need for more methadone programs outside the prisons, and the need for more halfway houses where patients could go after release as a transition point until they can become independent.

#### 3.2.2. Interviews with Prison Counselors (*n* = 8)

**Patients' Knowledge about HIV and Risk Reduction**

The counselor participants indicated that their patients are provided sufficient information about HIV, safe sex, and injecting practices but believed that they sometimes ignore the information provided, as exemplified by

*“People usually have the information about how to use a condom, not sharing needles… —before they are released—at least one day before release, but we do not know what happens after they are released” *and* “They know those things… we have told them about HIV… about protection, how to use it [condoms].” *

They all agreed that the patients tend to be relatively well informed while incarcerated. Counselors did report, however, that they are more concerned about prisoners' sex- and drug-risk behavior following release, as indicated by

*“[Following release], the prisoners are using condoms 20% of the time…” *and *“… So, the majority of them are sharing needles, because they have to, they… [feel like] they really need the drug… and they are still sharing cookers and other things.” *

**Patients' Attitudes about Risk Reduction Behavior**

When questioned about their patients' attitudes regarding risk reduction behavior, some responses were

*“When they are to have sex with a sex worker, the one that buys the condom is the sex worker not the guy, the client. So they don't know whether the brand is good or not” *and* “All the inmates love the wife. But sometimes, [they] don't want to use a condom because like if you want to use condom, then you have another person.” *

This implies that the counselor participants felt that a key barrier to engaging in condom use for their patients is that condom use may imply unfaithfulness to their spouse or primary partner and this may initially make them reluctant to use condoms with their primary partners. The counselor participants also reported that they believed their patients were also reluctant to use condoms because they felt that sex would be less pleasurable as exemplified by

*“… that's one of the problems, because my inmate also told me, eerr… he didn't want to use a condom because its [not satisfying].”*

Other responses regarding reluctance to use condoms were

*“If the inmate is a drug user who is HIV+, in his house he uses a condom, but outside he doesn't” *and *“… usually they do not have [unprotected] sex with their steady partner. Maybe he will satisfy his sexual needs in another manner” *and *“If it's a HIV positive sex worker, she's not interested. Who gave me the sickness? Why should I prevent someone else from getting it?… Yes, they don't care,” *

implying that some patients may be willing to protect their primary partners by using condoms, but not casual partners, and others may be harboring feelings of revenge.

Regarding drug-related behavior, some of the counselor participants' comments were

*“One inmate already came in last week and told me “who cares about HIV, who cares, who cares? I can share needles with others. The [only] certain thing is I need drugs now, now, now” and “Usually they don't care, they don't think. They don't think about it [HIV prevention]. They want drug, fun, high… When we see those with HIV, the question of prevention for them doesn't exist.”*

They all tended to agree that their patients' priority was to get high, and that they were less concerned with HIV transmission risk reduction. One counselor mentioned, for example, that it was not unusual for a patient to convince his spouse to go into sex work in order to support his drug habit.

When asked if addiction could be managed by methadone, on response was

*“If the patient is in a methadone program and he is really successful, then he will engage in the counseling program for methadone. Indirectly, the methadone program will make him concerned about other people.”*

They all agreed that methadone would enhance an HIV-risk reduction program.

**Intervention Content and Delivery Style**

Two of the counselor participants reported that they routinely demonstrate to their patients how to properly use condoms. Two other counselors reported not doing this. When asked about patients' skills in practicing safe sex, the counselor comments included

*“Not sure [if patients can properly put on condoms]”* and *“His sex, sexual desire… he doesn't care… because if he is high, he will sleep with anyone.” *

They, however, reported that they had been providing HIV information to their patients and teaching them risk reduction skills, as indicated by

*“We give the inmates information of course—every day—at least 4 times in one week, ok. We give them information. A new inmate, after 2 days, we give information about HIV, about AIDS, about CD4, then about needle sharing… about protected sex” *and *“I do tell them that just because you bought a condom doesn't mean you know how to use it properly. You must look at expiration date and know the proper way to use it.” *

Regarding their patients' ability to disclose their HIV status, counselor comments included

*“They don't know… how to tell [them]” and “When the inmates are scared to tell their wives about the infection, HIV, they will tell the counselor to contact their wives”*

Counselors tended to agree that their patients typically lack the skills to negotiate safe sex and the skills to disclose their HIV status to their families or others.

When asked about the barriers they have encountered, one counselor participant reported

*“In 1997, there was an Act released by the Prison Department that families must be informed of a confirmed HIV positive inmate's status. We have done this before, we called the family, [and] what arises here is a conflict. Inmates say that this is a confidential matter. I contact the family and ask them to come and meet me with the inmate. I focus more on [encouraging] the inmate to [tell the family], but what is happening now is some NGOs are questioning the confidentiality [in doing that].”*

Aside from confidentiality issues, one female counselor also mentioned that safety could be a barrier to discussing the details of sex-risk behavior because she did not feel safe discussing this topic with her male patients, as captured by

*“Because I'm a female, I am not comfortable when talking to a man inmate about condoms…. Very dangerous to talk too much with a man [like that].”*

There was also some concern that discussing sex a lot would give patients the license to practice it.

Overall, counselor participants were divided about whether intervention delivery would best be done in a one-on-one versus group format and saw advantages to each. One counselor indicated that, if one-on-one sessions were used, there should be gender matching to foster rapport and comfort between patients and counselors. When questioned about who would be the most feasible personnel to ultimately deliver this type of intervention, counselors all agreed that they were best positioned and qualified for this duty. Related to this role, counselors indicated that it would be important to develop a working manual and stressed the need for cooperation among all the directors and prison staff as exemplified by

*“For this to be more effective, we should have a manual. The manual will help the counselors. Not all of the counselors know about MET and CBT and other information. Not all [counselors] know. Manual will help and guide..and encourage us to be more confident.”* and *“To me, the most important thing is for the Directors of Institution to know about this program. If not, there is no cooperation, every level of prison staff should know.” *

#### 3.2.3. Interviews with Prison Correctional Officers (*n* = 4)

**Methadone Maintenance as Part of a Risk Reduction Strategy**

When asked about the potential role of methadone as part of a risk reduction program, some of the responses were

*“For me, the prisoners feel they have been given a chance [and] that we in the prison have given [them] a chance to be in a program that before was only done outside of prison. So, for me, his heart feels that he is not cast aside because we could run a program that was outside and is now in the prison” *and *“it can reduce dependency. It also will stop the prisoners from taking drugs… relapse. Since the program started here, from their behavior, I could see that they're not too addicted. And their behavior is better… before they were quite aggressive, but after methadone, their behavior is softer and less aggressive.” *

However, one participant added

*“For the inmates who are in the methadone program, their educational level is quite low. They don't quite understand the concept of rehabilitation. So the methadone program is really to take away the cravings but not as part of recovery/rehabilitation.”*

The correctional officer participants indicated that they do not maintain contact with released prisoners and therefore do not know the success rate of those who participated in the methadone program while in prison. Responding to whether there was the need for more information, one response was

*“The inmates need more awareness and education about methadone, its effects,… its benefits. The COs too needs awareness. Its better if we can get an outsider to come and give a talk.”*

When asked about the primary challenges that they face in implementing the methadone program, one response was

*“The inmates on methadone—for most of them—they would like to see a difference in a short time frame. They don't quite understand that to gain or to see a difference, it requires different doses. Usually we will explain to them repeatedly about the [dose] and effect.”*

There was also some discussion of the issues of side effects such as sleepiness and constipation.

**Intervention Content and Delivery Style**

When correctional officer participants were queried about the optimal content and delivery of a risk reduction intervention in the prisons, there was a tendency to favor a one-on-one session format, as indicated by

*“Giving information in group sometimes, you know people, there will distraction [from others]. So, when it is one-to-one, the information can be absorbed better. Understanding of the matter will be clearer.”*

It was suggested that the intervention content should be simple to understand, frequently reviewed, and should last no longer than one hour in order to improve attention, memory, and processing of the content.

Correctional officers agreed that laptop computers could be brought into the prison for these sessions as well as syringes, condoms, and replicas for demonstration purposes. It was also suggested that relevant information in the form of handouts and worksheets would be useful. In terms of meeting space, the correctional officer participants indicated that counseling rooms are typically available for one-on-one sessions as well as a 20-person conference room if a group format is preferable.

**Family Involvement following Release**

When questioned about the extent of family support for the target population, the correctional officer participants estimated that about half of the prisoners had disclosed their HIV status to their families. Regarding how to address this, one of the responses was

*“Counseling… Counseling on how to inform them and to encourage them. We can invite family here and provide an environment where inmates can start informing them.”*

The correctional officers stated that there tends to be a lot of variation in the responses that prisoners experience from their families, as exemplified by

*“It's all about the level of knowledge the families have about HIV. Families mostly know that it doesn't have a cure, it's deadly—you are going to die. There is also a lot of pressure from society on the families. Extended family—they need to make a judgment—do I support my child or my husband and have negative impact on society or I can stay away from my husband, child, etc. and continue living with my community? The prisoner is sometimes the [relationship] to be sacrificed” *and *“For some families, they can accept it, and for some they really can't… and you see the relationship is not as tight as it used to be.” *

Participants suggested that this situation may be reduced to a degree if the family had a better understanding of exactly how HIV is transmitted because misinformation is often part of the problem.

## 4. Intervention Refinement

Considered collectively, the formative research data obtained from prior prisoners, their families, and from treatment providers (medical doctors, counselor, and correctional officers) suggested that it would be most feasible to adapt the evidence-based Holistic Health Recovery Program for HIV-infected drug users (HHRP+ [21]), rather than attempting to implement it or any other existing EBI in the original form. We concluded that it was more practical to abridge the original HHRP+ intervention content to focus explicitly on HIV risk reduction and antiretroviral adherence, to place greater emphasis on certain interpersonal risk reduction skills (e.g., disclosure of HIV status, building healthier support systems including family, negotiating risk reduction with partners), and to redesign the delivery approach so that the intervention could be conducted in either a group or individual format depending on the needs of the inmate. Greater emphasis in the content was also placed on postrelease care (e.g., additional hands-on exercises designed to encourage participants to work through the specific steps required to connect with appropriate addiction and HIV care facilities after release) and support systems needed to foster such care. Equally important, we sought to preserve the style and process of the evidence-based HHRP+, while incorporating only the necessary modifications indicated by the elicitation data. 

In redesigning the intervention, we also considered an array of issues pertaining to the placement of the intervention within the context of the correctional system in Malaysia. This process was largely determined by the logistics of the target organizations as collectively described by the range of treatment provider participants. The key factors that we considered were (1) how the intervention could be made available to a maximum number of target participants, (2) how it could be positioned to be perceived by participants and staff as relevant to prisoners' overall clinical care, (3) how it could be the least disruptive to the organization's routine, and (4) how it could be placed so that it would be most likely to be sustained over time, as designed, using the existing human and physical resources available within the correctional system in Malaysia. The resulting intervention, the Holistic Health Recovery Program for Malaysia (HHRP-M; Table 4) consists of eight 2-hour sessions designed to cover a range of relevant topics so that each participant may choose to apply intervention content as needed to their own HIV risk profile and antiretroviral adherence challenges. Importantly, the HHRP-M intervention manual was deliberately designed to be sufficiently flexible so that content can be readily delivered in a group or individual format and so that sessions can be delivered in either consecutive or weekly meetings based on logistics and time constraints. It was also designed to encourage participants' concurrent enrollment in drug treatment and HIV care as complementary risk reduction and relapse prevention strategies and in order to enhance the likelihood that inmates will be able to make a healthier transition back to the community.

## 5. Disscusion

This study involves a description of the process and outcome of formative research that was conducted in preparation to adapt an evidence-based intervention (EBI) for use with drug-involved HIV-infected soon-to-be-released prisoners in Malaysia. The Holistic Health Recovery Program for Malaysia (HHRP-M) is a behavioral intervention that has been designed to address the HIV risk behavior and HAART adherence challenges faced by HIV-infected inmates in the correctional system in Malaysia as they transition back to the community. 

The results of our study suggest that there is a great need for sex- and drug- risk harm reduction interventions in these Malaysian prisons and that current and, soon-to-be-released prisoners will benefit significantly from such an intervention. It is evident that there is much room for improvement in the target populations' level of information about HIV and risk reduction, and in their motivation and skills to practice healthy behaviors and these were the major focus in designing the HHRP-M. For example, the report of sharing injecting paraphernalia with other infected individuals shows a gap in the area of reinfection and superinfection. Also, the significant decline or complete absence of healthcare in the after reentering the community can have very severe implications for HIV-positive individuals suggesting the need to begin an intervention prior to their release.

Our results also show an almost unanimous positive disposition toward methadone maintenance and its potential role in harm reduction and this was observed in all groups of participants. Similar findings have been observed in other studies [25]. A study in Taiwan [26] showed that methadone maintenance was associated with reduced mortality, independent of HIV status, in a cohort of newly released prisoners who had a history of injection drug use. The role of methadone maintenance in harm reduction has also been suggested in several studies [27, 28].

Although several barriers to addressing this intervention were raised, these are issues that could be tackled in the adaptation process. Wiley's global framework [22]—which includes accommodation, incorporation, and adaptation—was used as an overarching approach to intervention adaptation. Our specific adaptation approach was consistent with the Assessment-Decision-Administration-Production-Topical experts-Integration-Training-Testing (ADAPT-ITT) model [23] in which input from the target population and treatment providers was systematically elicited and then incorporated into the refinement of an evidence-based intervention. Information gleaned from these multiple sources had a significant impact on the features of the resulting HHRP-M intervention, including the emphasis of certain content (e.g., family/social support; planning for post-release treatment) and the flexibility of intervention delivery. This information was also critical in terms of informing intervention refinement such that the intervention was perceived as engaging and relevant by participants and treatment providers as well as realistic in terms of the organizational characteristics common in the correctional facilities in Malaysia. For example, overcoming stigma is a feature of the intervention that will be very useful in this setting as evidenced by the reluctance of some patients to disclose their HIV status, and the reaction of some families to their relative's HIV status. Prior research has demonstrated that proactively addressing such issues reduces the need to make major adjustments to an intervention in the midst of implementation [20]. 

The objective of this study was to obtain qualitative information that could be useful in designing an optimal adapted evidence-based intervention approach for use in correctional settings in Malaysia and this resulted in the limitations that are inherent in research with a qualitative design. We believe, however, that our careful selection of participants, a well-established analytical approach, and the incorporation of published empirical research, resulted in well-informed and empirically justified decisions that were used to drive the adaptation process. Our adapted intervention, HHRP-M, is currently being deployed in a large RCT in correctional settings in Malaysia and has yet to be evaluated in terms of quantitative outcomes. Thus, while there are strong indications of the feasibility of incorporating HHRP-M in such settings, it is not yet possible to draw conclusions regarding the intervention's efficacy in this or similar settings.

Despite the noted limitations, this study points to the potential benefits of systematically adapting culturally responsive intervention approaches across international contexts by taking into consideration a range of overarching cultural issues as well as the very specific issues unique to particular clinical setting(s) such as prisons where high risk populations may be readily reached. A secondary objective of reporting our formative research process and outcomes was to inform similar efforts in the future as a growing number of EBIs have become widely available to interventionists (see http://www.effectiveinterventions.org/) yet such interventions are not necessarily in optimal form for implementation in clinical settings without proper adaptation.

## Figures and Tables

**Table 1:** Instrument for interviews with target population and family members.

Questions for target population	(1) When you think about problems or concerns that are important in your life right now, which ones are most important? What do you worry about the most? (2) Did you receive HIV information in prison? Was it helpful? (3) Tell me about the ways HIV can be transmitted (4) Tell me how HIV can be prevented (5) Do you think that prisoners are having sex and injecting drugs, and if so, are they safe? (6) Do you like condoms? What do you like/not like about condoms (7) Do you share needles or do you clean needles or get new needles? (8) What has been your experience with methadone? (9) What type of intervention would work best (individual or group)? (10) How long should each session last? (11) Is your family aware of your HIV status? (12) Is it good to involve your family in an intervention before your release? (13) What can or cannot be discussed with family present?

Questions for family members	(1) Do you worry a lot about your family member being HIV+? What is it about them being HIV+ that worries you? Did you learn about it (HIV status) directly from your family member or from someone else? (2) Do you have any fears or concerns about HIV being transmitted to anyone in your family just by living in the same house or having meals together? (3) Do you take any special precautions when your family member is around to keep HIV from being transmitted? (4) Did you or other members of your family go to visit your family member while s/he was in prison? Did you place any conditions on whether s/he could come to live with you upon release? (5) What do you think causes someone to use drugs? (6) What do you tell your family member in order to get him/her to stop using drugs? Has it ever worked? (7) How do you think families *help* or *hinder* a person from stopping using drugs? (8) What do you think about methadone or buprenorphine? What are the positive things about it and what are the negative things? (9) Do you think that programs for HIV education are helpful? What would be the most helpful?

**Table 2:** Interview instrument for treatment providers.

General questions for all treatment providers	(1) Do you think your patients have sufficient information on HIV? (2) What types of HIV risk behaviors (sex- and drug-related) do you perceive in these patients? (3) Why do patients continue to practice risky behaviors? (4) Have your patients disclosed their status to their families? (5) How do you feel about the methadone program? (6) What type of intervention would work best (e.g., individual, group)? (7) How long should each session last? (8) What materials can or cannot be brought into the prison for the purpose of the intervention?

Questions for physicians	(1) Do you counsel your patients on HIV risk reduction? (2) Are you uncomfortable administering methadone? (3) What are the challenges you have experienced (with methadone)? (4) What are the side effects of methadone your patients have complained about? (5) What suggestions do you have improving the continuity of care (e.g., methadone therapy and HIV medications) after release?

Questions for counselors	(1) Do you think there are information deficits that may lead to risky behavior? (2) Do you think there may be any motivational obstacles that contribute to this risky behavior? (3) What negative attitudes toward safer sexual and drug behaviors do patients possess? (4) What norms do patients have that interfere with safer sexual and drug using behaviors? (5) Do you think there may be any deficits in behavioral skills that may contribute to any risky behavior? (6) What has been the reaction from their families and how can we make it easier to disclose? (7) What approaches do you use right now that may be helping to increase patients' HIV preventive behaviors? (8) Will you be comfortable discussing sensitive issues like sex and condom use with patients? (9) What mode of presentation would work best (psychoeducational, power-point, handouts)? (10) What kinds of support could be provided to help you integrate the intervention?

**Table 3:** Demographic characteristics of all interview participants.

	Target population (*n* = 8)	Family members (*n* = 3)	Prison doctors (*n* = 5)	Prison counselors (*n* = 8)	Prison correctional officers (*n* = 4)
Gender	Male (8)	Male (1)	Male (5)	Male (6)	Male (4)

Age	28–48	Not available	44–64 (mean 55.8)	Not available	33–45 (mean 39)

Ethnicity	Malay (8)	Malay (3)	Indian (2) Burmese (2) Chinese (1)	Malay (8)	Malay (4)

Prison facility	Pengkalan Chepa (8)	n/a	Kajang (1) Penang Island (1) Pokok Sena (1) Penor (1) Kluang (1)	Kajang (1) Kluang (1) Penor (1) Penang Island (1) Pengkalan Chepa (2) Pokok Sena (1) Seremban (1)	Pengkalan Chepa (4)

Yrs work experience	n/a	n/a	Mean 1.6	Range = 3–15 (mean 7.4)	Range = 4–6 (mean 5.3)

**Table 4:** Outline of adapted intervention sessions.

Group topic	Information, motivation, and skills taught
(1) Health care participation	Actively participating in your health care, Improving skills for partnering with health care providers; Building ARV adherence skills; HIV and opportunistic infections.

(2) Reducing drug and sex-related risk and negotiating RR with partners	Harms of injecting drugs & harm reduction techniques (e.g., proper needle cleaning); Reducing cue-elicited craving, Harms of unsafe sexual practices & Safer sex techniques (e.g., condom applications); Building skills to communicate about risk reduction with your partner.

(3) Healthy lifestyle choices	Learning coping skills; Stress management skills; Building skills to identify and plan healthy daily activities; Learning & practicing several relaxation techniques; Building skills to identify and plan healthy daily activities

(4) Preventing relapse to risky behavior: Recovery as a journey	Building a road map for the journey of recovery; Relapse prevention skills; Identify early warning signs; Understanding and managing seemingly irrelevant decisions (SID).

(5) Overcoming stigma	Understand stigma and consequences; Decreasing the strength of the “addict” self & connecting with the “core” self; Identifying and strengthening the cognitive, affective, and behavioral attributes of a healthier, non-drug using lifestyle; Building skills to redefine the self as a non-drug user.

(6) Motivation for change; overcoming helplessness	Understanding the sources of helplessness and the consequences; Identify empowering situations; Build skills to assess readiness to change; Increase motivation to pursue a healthy lifestyle.

(7) Moving Beyond Grief	Understanding the stages of grief; Build skills to cope with fears related to HIV; Facing fears and reclaiming control of life; Identifying and prioritizing that which has personal meaning.

(8) Reaching your goals	Learning about memory and concentration and how to improve; Build skills to set goals, establish priorities, and initiate action; Building skills to improve nutirition pyramid; Developing medication adherence skill.
